# A new method for the rapid detection of the antibacterial and bacteriostatic activity of disinfectants based on Propidium Monoazide combined with real-time PCR

**DOI:** 10.3389/fmicb.2022.1051162

**Published:** 2022-11-08

**Authors:** Yanrong Liu, Shuting Huang, Jungui Zhou, Chi Zhang, Feijie Hu, Youyu Xiao, Haopu Qiu, Yao Yang

**Affiliations:** ^1^Key Laboratory of Biotoxin Analysis and Assessment for State Market Regulation, Nanjing Institute of Product Quality Inspection, Nanjing, China; ^2^School of Food Science and Pharmaceutical Engineering, Nanjing Normal University, Nanjing, China

**Keywords:** disinfectants, antibacterial and bacteriostatic activity, PMA-qPCR method, homogenization method, rapid detection

## Abstract

Rapid detection of antibacterial and bacteriostatic properties is an important part of the quality and safety supervision of disinfectants. In this study, propidium monoazide (PMA) was used in combination with real-time PCR (PMA-qPCR) to detect the antibacterial and bacteriostatic activity of disinfectants against three commonly used indicator bacteria, *Escherichia coli*, *Staphylococcus aureus*, and *Candida albicans*, utilizing specifically designed primers. The method for preparing membrane-damaged bacteria was optimized to improve the ability of the PMA dye to distinguish between live and dead indicator bacteria. Finally, this method could simultaneously detect viable numbers of the indicator bacteria after the disinfectants were used. The *R*^2^ values of the PMA-qPCR standard curves were 0.9986, 0.9980, and 0.9962 for *E. coli*, *S. aureus*, and *C. albicans*, respectively, and the detection range was 10^3^ ~ 10^6^ CFU/ml, showing no significant difference in accuracy compared to that of the plate counting method (*p >* 0.05). The method established here is the first application of PMA-qPCR to detect the antibacterial and bacteriostatic activity of disinfectants. This technique markedly simplifies the detection steps of antibacterial and bacteriostatic activity, reduces the detection time (3 h compared to 48 ~ 72 h for the plate counting method), improves the quality supervision efficiency of disinfectants, and guarantees healthy and safe lives.

## Introduction

The novel coronavirus has caused a worldwide infection since its outbreak in 2019, and the situation remains dire. The market share of disinfectants has proliferated to maximize people’s health status; however, their quality control is facing an unprecedented challenge. Disinfectants products include disinfectant solution, disinfection devices (including biological indicators, chemical indicators, and packaging for sterilized items), hygiene products, and single-use medical supplies (Fathizadeh et al., 2020). Disinfectants are widely used to ensure the quality and safety of medical devices, human life, and drinking water. Supervision of the product quality and safety of disinfectants, especially research on the rapid detection methods of antibacterial and bacteriostatic activity of disinfectants, is an important scientific issue related to health and the harmonious development of society (Rutala and Weber, 2016).

Disinfection refers to the elimination, removal, and suppression of pathogens and other harmful microorganisms in the environment. The evaluation of the effectiveness of disinfectants often relies on laboratory cultures because of the invisibility of microorganisms to the naked eye. The current national standards for the antibacterial and bacteriostatic activity of disinfectants are based on the indicator bacteria plate count method (China Technical Standard For Disinfection 2002), with the following main indicators: *Escherichia coli* 8099 stands for enteric bacteria, *Staphylococcus aureus* ATCC 6538 stands for septic cocci in bacterial colonies, and *Candida albicans* ATCC 10231 stands for pathogenic fungi. The specific method of this assay is to perform a plate count of the indicator bacteria from the treated samples and calculate the bactericidal inhibition rate from the difference in the number of viable bacteria compared to that of the control samples. The plate count method is simple, but the number of steps and workload is large, the turnaround time for test results is approximately 48 h (bacteria) to 72 h (yeast), and many sublethal indicator bacteria cannot form colonies because of the limitations of the culture environment (Kumar and Ghosh, 2019). Therefore, the plate count method cannot provide rapid evaluation of the antibacterial and bacteriostatic performance of disinfectants.

In recent years, real-time PCR (qPCR) has been widely used for the rapid quantitative detection of microorganisms (Kumar and Ghosh, 2019). The principle of this method is to amplify specific target microorganism genes by designing fluorescent dyes or fluorescence-labeled specific primers, and then determining the number of microorganisms in the sample by quantifying the initial template of the PCR reaction. The qPCR method, a detection technique that does not depend on microbial culture, drastically reduces the detection time; however, the nucleic acid detection method does not distinguish between dead and live bacteria (Zhao et al., 2017). Therefore, conventional qPCR methods are limited in the detection of the antibacterial and bacteriostatic activity of disinfectants.

Ethidium monoazide (EMA) and propidium monoazide (PMA) are a class of photoreactive dyes with a high affinity for DNA (van Frankenhuyzen et al., 2011). They are embedded in double-stranded DNA under intense visible light to form a covalently linked chemical modification that cannot be amplified by PCR. Because EMA and PMA are completely impermeable to cell membranes, this property allows them to be used in combination with qPCR to distinguish between dead and live bacteria. Recently, researchers have found that EMA treatment causes the loss of genomic DNA in live bacteria; therefore, PMA dyes have been chosen more often for this type of study (van Frankenhuyzen et al., 2011). Currently, PMA dye combined with fluorescent qPCR (PMA-qPCR) is widely used as a rapid detection method in food, medicine, and the environment. It detects not only bacteria, such as *E. coli* (Miotto et al., 2020; Deshmukh et al., 2021), *Salmonella* (Techathuvanan and D'Souza, 2020), *Lactobacillus* (Yang et al., 2021), *Vibrio* (Copin et al., 2021), and *Listeria monocytogenes* (Kragh et al., 2020), but also fungi, such as *C.albicans* (Asadzadeh et al., 2018), and even virus (Zeng et al., 2022).

The present study is the first application of PMA dye combined with qPCR for the rapid detection of the antibacterial and bacteriostatic activity of disinfectants. In contrast to the existing PMA-qPCR method, by optimizing the conditions, we aimed to develop a reaction system in which one product to be tested simultaneously acts on multiple indicator bacteria and completes the specific quantitative detection of multiple live indicator bacteria. This method may significantly improve the detection efficiency of the antibacterial and bacteriostatic activity of disinfectants and provide a practical basis for PMA-qPCR for the detection of live bacteria in mixed samples.

## Materials and methods

### Microorganisms and culture conditions

The bacterial strains and the culture media used in this study are listed in Table 1. The indicator strains were grown in the corresponding culture medium (Table 1).

**Table 1:** Reference strains and specific primers verified by qPCR.

Strain	Culture medium	Culture temperature	Ct values		
			Primer of EC	Primer of SA	Primer of CA
*Escherichia coli* ATCC 8099	Nutrient agar	36°C	15.08 ± 0.15	–	–
*Staphylococcus aureus* ATCC 6538	Nutrient agar	36°C	–	14.64 ± 0.50	–
*Candida albicans* ATCC 10231	Sabouraud’s agar	36°C	–	36.11 ± 0.17	9.08 ± 0.15
*Enterobacter aerogenes* ATCC 13048	Nutrient agar	36°C	35.64 ± 0.18	–	–
*Staphylococcus epidermidis* CMCC(B)26,069	Nutrient agar	36°C	–	35.92 ± 0.12	–
*Salmonella* ATCC 14028	Nutrient agar	36°C	35.28 ± 0.34	36.11 ± 0.03	35.13 ± 0.25
*Pseudomonas aeruginosa* ATCC 27853	Nutrient agar	36°C	–	–	–
*Pseudomonas fluorescens* ATCC 13525	Nutrient agar	36°C	–	–	–
*Bacillus subtilis* ATCC 6633	Nutrient agar	36°C	36.21 ± 0.54	36.77 ± 0.14	34.71 ± 0.56
*Listeria monocytogenes* ATCC 19111	BHI agar	36°C	–	–	–
*Saccharomyces cerevisiae* ATCC9763	Sabouraud’s agar	36°C	35.31 ± 0.31	36.17 ± 0.12	34.86 ± 0.16

“–” indicates that no band is amplified.

### Species-specific general primers and Probes designed

Specific primers were designed based on the gene sequences of the three indicator bacteria, and the corresponding probe primers were synthesized to improve the specificity of detection of the target indicator bacteria. The primers for amplifying the target sequences of *E. coli* were named EC-F, EC-R, and EC-P, the primers for amplifying the target sequences of *S. aureus* were named SA-F, SA-R, and SA-P, and the primers for amplifying the target sequences of *C. albicans* were named CA-F, CA-R, and CA-P. The results are listed in Table 2. The primer probes were synthesized by Bioengineering (Shanghai).

**Table 2:** The primer used in this study.

Name of the primer	Primer sequence	Product size	Reference gene	References
EC-F	5′-gcgggtatttggctacgtaacga-3’	109 bp	*lac*Y	Yuan et al. (2018)
EC-R	5′-ccagcagcagggcatttttc-3′			
EC-P	5’-VIC-tgcgccactgatcat-MGB-3′			
SA-F	5′-tagggatggctatcagtaatgttt-3′	105 bp	GenBank ID: DQ507382.1	Zhang et al. (2015)
SA-R	5′-ctatttacgccgttacctgtttgt-3′			
SA-P	5’-CY5-agaacaatacacaaagagg-MGB-3′			
CA-F	5′-cagaagtgacaggaacagcaatca-3′	94 bp	*sap*I	Lin et al. (2021)
CA-R	5′-gccactggacaaatcattttcg-3′			
CA-P	5’-FAM-ccactgtatttagctttgtca-MGB-3′			

### DNA extraction

For bacterial DNA extraction, cells were first lysed by the homogenization method and then extracted using phenol:chloroform:isopentyl alcohol (25,24,1), which is consistent with our previous reports (Yang et al., 2021). The DNA concentration was measured using a microspectrophotometer.

### Quantitative qPCR analysis

The qPCR reaction system was (20 μl):2× SYBR Premix ExTaq 10 μl, 0.4 μl each of upstream and downstream primers and probe primer (10 μmol·L-1), 2 μl of template DNA (10 ng·μL-1), and dd H2O was added to 20 μl. A negative control reaction was set without DNA.

The amplification conditions were: pre-denaturation at 95°C for 30 s, denaturation at 95°C for 5 s, annealing at 58°C for 30 s, and extension at 72°C for 30 s for 40 cycles. The fluorescence signal was collected at the time of warming to establish a melting curve.

### Specificity and sensitivity of qPCR

Primer specificity was verified by extracting genomic DNA from the three experimental strains and eight reference strains mentioned above, amplifying the samples by qPCR using the three primers mentioned above, and determining the specificity of the primers based on the results of the amplified Ct values (Zhai et al., 2019).

Primer amplification efficiency was verified by taking 1 ml of the prepared experimental broth with OD600 ≈ 1 (refer to live cells at the beginning of the exponential phase), and the number of viable bacteria in the experimental broth was determined by plate counting (Kragh et al., 2020). At the same time, whole genomic DNA was extracted, and gradient dilutions (10^1^ to 10^6^ dilutions) were prepared to obtain samples with different initial DNA concentrations. The samples were amplified by qPCR using the above primers, and the linear equation of the Ct value versus initial DNA concentration was plotted to calculate the qPCR amplification efficiency E value (E = 10^-1/slope^). Three replicates were performed for each qPCR sample.

### Killing method for obtaining the dead cells of indicator bacteria

#### Heating methods

One milliliter of the experimental bacterial solution with OD600 ≈ 1 prepared as above was placed in a 1.5 ml centrifuge tube, washed twice in phosphate buffer solution (PBS), and resuspended in 1 ml of PBS buffer. The bacteria were treated in a water bath at 100°C for 15 min for heat lethality, and the untreated suspension was used as the control group. Three replicates were performed for each sample.

#### Homogenization (mechanical) method

One milliliter of the experimental bacterial solution with OD600 ≈ 1 prepared as described above was placed in a 1.5 ml centrifuge tube, washed twice in PBS buffer, and resuspended in 1 ml of PBS buffer. The homogenization program was set to a homogenization speed of 6.0 m/s, working time of 30 s, and interval of 30 s. A total of 15, 20, 25, 30, and 35 cycles were performed, with the untreated bacterial suspension as the control group. Three replicates were performed for each sample.

The samples obtained using the above method were directly coated with 200 μl of the corresponding solid medium plates and incubated at 37°C for 24 ~ 48 h. These results were used to determine the effectiveness of the preparation of membrane-damaged bacteria. Three replicates were performed for each sample.

### PMA treatment for PMA-qPCR

The working concentration of PMA dye-treated samples (experimental bacterial solution or samples to be examined) in this study was 40 μg/ml (Zhao et al., 2019; Yang et al., 2021). The PMA was well mixed with the samples, incubated for 5 min protected from light, followed by 15 min of light reaction time, and the PMA-treated samples were analyzed in a PMA-Lite LED Photolysis Device (BIOTIUM, E90002, USA).

### Construction of PMA-qPCR standard curve

One milliliter of the experimental bacterial solution with OD600 ≈ 1 prepared by the above method was collected by centrifugation at 12,000 × *g* for 1 min, washed twice with PBS buffer solution, and resuspended in an equal volume of PBS buffer solution. The suspensions were divided equally into two groups: one group without any treatment, that is, the live group, and one group with membrane-damaged bacteria prepared first according to the optimized method, followed by PMA treatment. DNA was extracted from both groups, and the DNA of the live group was diluted in a gradient (10^0^ ~ 10^6^ times) with the DNA of the membrane-damaged group. The Ct values were obtained by qPCR amplification using the designed primers. Ct values were obtained using an ABI 7500 FAST fluorescent quantitative PCR instrument. Three replicates were performed for each sample. A standard curve of Ct values versus the initial DNA concentration of live bacteria after PMA treatment was plotted.

### Testing of disinfection products

The disinfectant products tested were: Refreshing Hand sanitizer, produced by Shanghai Jahwa United for germicidal experiments; Willows Foam Antibacterial Hand sanitizer, produced by Willis (Guangzhou) Household Products for germicidal experiments; Jierou sanitary wipes, produced by Zhongshun Jierou (Sichuan) Paper for sterilization experiments; Vida sanitary wipes, produced by Vida Paper (Beijing) for germicidal experiments; 84 disinfectants, produced by Jiangsu Atef 84 for sterilization experiments; and Hand disinfectant, produced by Nanjing Zhuhai Biotechnology for sterilization experiments.

#### Handwash inhibition test

A 10 g sample of hand sanitizer was weighed, added to an equal mass of PBS (0.03 mol/l, pH 7.2), homogenized, and prepared to obtain the sample solution to be tested.

Plate counting detection refers to China Standard GB15979-2002: Hygienic standard for disposable sanitary products. The PMA-qPCR method was slightly modified on the basis of the plate counting method, as follows: the 24 h slant culture of a single indicator bacterium was washed with PBS to make a bacterial concentration of approximately 5 × 10^5^ ~ 4.5 × 10^6^ CFU/ml suspension; the three indicator bacterial suspensions were mixed in equal volumes; 300 μl of the mixed suspension was added to 5 ml of the sample solution, and 300 μl of the control sample was added to 5 ml of PBS for the control group. After 2 min, the experimental and control samples (0.5 ml) were placed in a test tube containing 5 ml of PBS and mixed well to terminate the inhibition experiment. The experimental and control samples were treated with PMA, and DNA was extracted and subjected to qPCR assays. The Ct values were recorded, and the number of viable bacteria in the sample solution was calculated using the PMA-qPCR standard curve.

#### Germicidal test of sanitary wipes

The neutralizing agent was identified according to China Standard GB15979-2002: Hygienic standard for disposable sanitary products, and the neutralizing agent of the sanitary wipes used in this study was determined to be “Tryptic Soybean Peptone Liquid Medium (TSB) containing 1% sodium thiosulfate and 1% Tween 80.” The PMA-qPCR method was modified slightly by mixing three indicator bacterial suspensions in equal volumes, each with a concentration of 5 × 10^5^ ~ 4.5 × 10^6^ CFU/ml. Next, 300 μl of the indicator bacterial suspension was added dropwise to the control sample. After the reaction was terminated by the neutralizer, the samples of the experimental group and the control group were treated with PMA, and DNA was extracted and subjected to qPCR. Ct values were recorded, and the number of viable bacteria in the samples was calculated using the PMA-qPCR standard curve.

#### Sterilization experiments with disinfectants

The neutralizer identification test was performed according to China Technical Standard For Disinfection 2002, and the neutralizer used in this study for the 84 disinfectant samples was PBS containing 0.5% sodium thiosulfate, 0.2% lecithin, and 2% Tween 80″. The disinfectant sample to be tested was prepared using sterile hard water at a concentration of 1.25 times the concentration to be tested.

The PMA-qPCR method was slightly modified in the experimental method, and the indicator bacterial suspension was mixed with three types of suspensions in equal volumes, and the concentration of each indicator suspension was approximately 3 × 10^8^ CFU/ml to 1.5 × 10^9^ CFU/ml. The samples were treated with PMA, and DNA was extracted and subjected to qPCR. Ct values were recorded, and the number of viable bacteria in the samples was calculated using the PMA-qPCR standard curve.

### Statistical analysis

The experiments were repeated three times with all indicators in three parallel groups. The results are expressed as x ± s. The SPSS software (version 2.0) was used for the statistical analysis of the experimental data. The two groups were analyzed using independent samples *t*-test, and the significance level was set at 0.05.

## Results

### Primer specificity validation

The specificity of qPCR primers was verified according to a previously described method. The qPCR results obtained for the Ct values are shown in Table 1. DNA from the three target indicator bacteria were successfully amplified using the corresponding primers, with Ct values ranging from 9.08 ± 0.15 to 15.08 ± 0.15 (Table 1). Moreover, amplification of other indicator bacteria and reference strains using the target primers was unsuccessful, illustrating the specificity of the qPCR primers.

### Validation of primer amplification efficiency

The experimental bacterial solutions prepared at OD600 ≈ 1 for the indicator bacteria were subjected to plate colony counting. The results showed that the experimental concentrations of the three different indicator bacteria, *E. coli*, *S. aureus*, and *C. albicans*, were 1.1 × 10^9^, 5.6 × 10^8^, and 4.5 × 10^8^ CFU/ml, respectively. The genomic DNA of the three indicator bacteria was then extracted separately, diluted in a gradient to obtain 10^2^ ~ 10^9^ CFU/ml bacterial DNA concentrations, and used sequentially as templates for qPCR amplification. The Ct values of the single indicator bacteria were plotted against a standard curve of log_10_ CFU/mL, and the experimental results are shown in Table 3.

**Table 3:** The amplification efficiency of qPCR.

Indicator bacteria	Equation of linear regression	*R*^2^ value	Detection range	*E* value^a^
			(log_10_CFU/mL)	
*Escherichia coli*	*y* = −3.4571x + 37.1901	0.9993	3 ~ 6	94.65%
*Staphylococcus aureus*	*y* = −3.7889x + 42.3801	0.9995	3 ~ 6	93.62%
*Candida albicans*	*y* = −3.1250x + 36.4680	0.9991	3 ~ 6	92.95%

^a^*E*-value = 10^(−1/slope)^.

The results showed that DNA from the three indicator bacteria was successfully amplified using the corresponding qPCR in the range of 10^3^ ~ 10^6^ CFU/ml, and the amplification efficiency was in the range of 90 ~ 105%.

Based on these results, we concluded that the qPCR primers had good specificity and high amplification efficiency. To further improve the detection efficiency, three indicator bacteria primers were added to the qPCR system simultaneously in the actual disinfectant sample testing to achieve a one-step detection of different indicator bacteria.

### Preparation of membrane-damaging bacteria

#### Heating method

The three indicator bacteria were treated separately in a 100°C water bath for 15 min, and the samples before and after treatment were subjected to plate counting and qPCR. In addition, the heated samples were subjected to PMA treatment and subsequent qPCR analysis; the results are shown in Table 4.

**Table 4:** Experimental results of the heating treated samples.

Indicator bacteria	Plate count		qPCR		PMA-qPCR
	(log_10_CFU/mL)		(Ct value)		(Ct value)
	Before heating	After heating	Before heating	After heating	After heating
*Escherichia coli*	8.87 ± 0.57	–	11.92 ± 0.17	12.62 ± 0.34	20.66 ± 0.43
*Staphylococcus aureus*	6.14 ± 0.35	–	12.62 ± 0.68	18.86 ± 0.21	20.17 ± 0.28
*Candida albicans*	4.14 ± 0.46	–	14.37 ± 0.19	19.24 ± 0.38	21.11 ± 0.37

“–” indicates that no clone growth.

The heating method was effective for fragmenting all three indicator bacteria, and no colonies grew on the corresponding plates after heating (Table 4). However, qPCR showed that the Ct values changed before and after the heat treatment, especially for S. aureus and C. albicans. The ΔCt values were 6.24 and 4.87 before and after treatment, respectively, which is highly variable, and it was seen that the heat treatment caused different degrees of damage to the DNA of all three indicator bacteria, which affected the amplification of PCR reactions. Although the Ct values of qPCR were further reduced after PMA treatment, it could not be determined whether heat treatment influenced the PMA-bound DNA. Therefore, the heating method is not applicable for the preparation of membrane-damaged bacteria. Currently, the heating method has been used in many PMA-qPCR studies for the preparation of membrane-damaged bacteria; however, optimization of the heat treatment conditions needs to be performed according to different target strains. Because the goal of the current study was to detect three indicator bacteria simultaneously, the generality of membrane-damaged bacterial preparations was required; therefore, no further attempts to optimize the heat treatment method were made.

#### Homogenization (mechanical) method

To determine the appropriate homogenization intensity that could simultaneously target the three indicator bacteria, a one-way experiment on the number of homogenization cycles was conducted, and the results are shown in Table 5.

**Table 5:** Optimization results of the homogenization method.

Indicator bacteria	Cycles				
Plate counting result					
(log_10_CFU/mL)	15	20	25	30	35
*Escherichia coli*	–	–	–	–	–
*Staphylococcus aureus*	4.58 ± 0.12	2.83 ± 0.28	1.73 ± 0.25	–	–
*Candida albicans*	4.08 ± 0.34	2.64 ± 0.23	1.92 ± 0.18	1.62 ± 0.26	–

“-” indicates that no clone growth.

The homogenization method did not have consistent fragmentation effects for the three different indicator bacteria (Table 5). The gram-negative bacterium *E. coli* has a sparse cell wall, is easily fragmented, and was completely fragmented after 15 homogenization cycles, whereas the gram-positive bacterium *S. aureus* and *C. albicans* required increased mechanical fragmentation intensity owing to their dense cell walls. According to the experimental results, the final condition of the homogenization crushing treatment was determined as 6.0 m/s (work 30 s and stop 30 s) per cycle for 35 cycles.

The three indicator bacteria were homogenized and separately fragmented. The samples were subjected to qPCR before and after treatment, and homogenized samples were subjected to PMA and subsequent qPCR analyses. Table 6 presents the results.

**Table 6:** qPCR results of homogenization treated samples.

Indicator bacteria	qPCR		PMA-qPCR	ΔCt^a^
	(Ct value)		(Ct value)	
	Before homogenization	After homogenization	After homogenization	Before and after PMA treatment
*Escherichia coli*	11.84 ± 0.13	9.82 ± 0.33	24.67 ± 0.23	12.83
*Staphylococcus aureus*	12.43 ± 0.58	12.48 ± 0.19	25.16 ± 0.21	12.73
*Candida albicans*	14.07 ± 0.16	14.21 ± 0.28	25.17 ± 0.47	11.1

^a^ΔCt = Ct value of PMA-qPCR - Ct value of the control group.

The Ct values of qPCR before and after homogenization of the three indicator bacteria were not significantly different, which revealed that this treatment did not affect the amplification of genomic DNA of the strains involved in subsequent PCR reactions (Table 6). In addition, the Ct values of qPCR after PMA treatment were significantly lower than those before PMA treatment, with ΔCt values of 12.83, 12.73, and 11.10 for *E. coli*, *S. aureus*, and *C. albicans*, respectively, indicating that PMA could distinguish between live and membrane-damaged bacteria. These results fully illustrated the feasibility of PMA-qPCR for the quantification of viable bacteria among the three indicator bacteria.

### Construction of PMA-qPCR standard curve

Standard curves of PMA-qPCR for the three indicator bacteria were established according to the methods described above (Table 7). The results show that the standard curves had a good linear relationship, and all of the concentration ranges of the detected *E. coli*, *S. aureus*, and *C. albicans* were 10^3^ ~ 10^6^ CFU/ml, respectively, which met the requirements for antibacterial and bacteriostatic effects of disinfectants (China Standard GB15979-2002: Hygienic standard for disposable sanitary products).

**Table 7:** PMA-qPCR standard curves of three indicator bacteria.

Indicator bacteria	Equation of linear regression	*R*^2^ value	Detection range
			(log_10_CFU/mL)
*Escherichia coli*	*y* = −3.3559x + 36.7329	0.9986	3 ~ 6
*Staphylococcus aureus*	*y* = −3.238x + 38.8192	0.998	3 ~ 6
*Candida albicans*	*y* = −3.168x + 36.5982	0.9962	3 ~ 6

### Testing of disinfection products

The antibacterial and bacteriostatic activity of the three disinfectants were tested using the PMA-qPCR method and were compared with those of the corresponding national standards adopted for plate counting.

**Figure 1:**
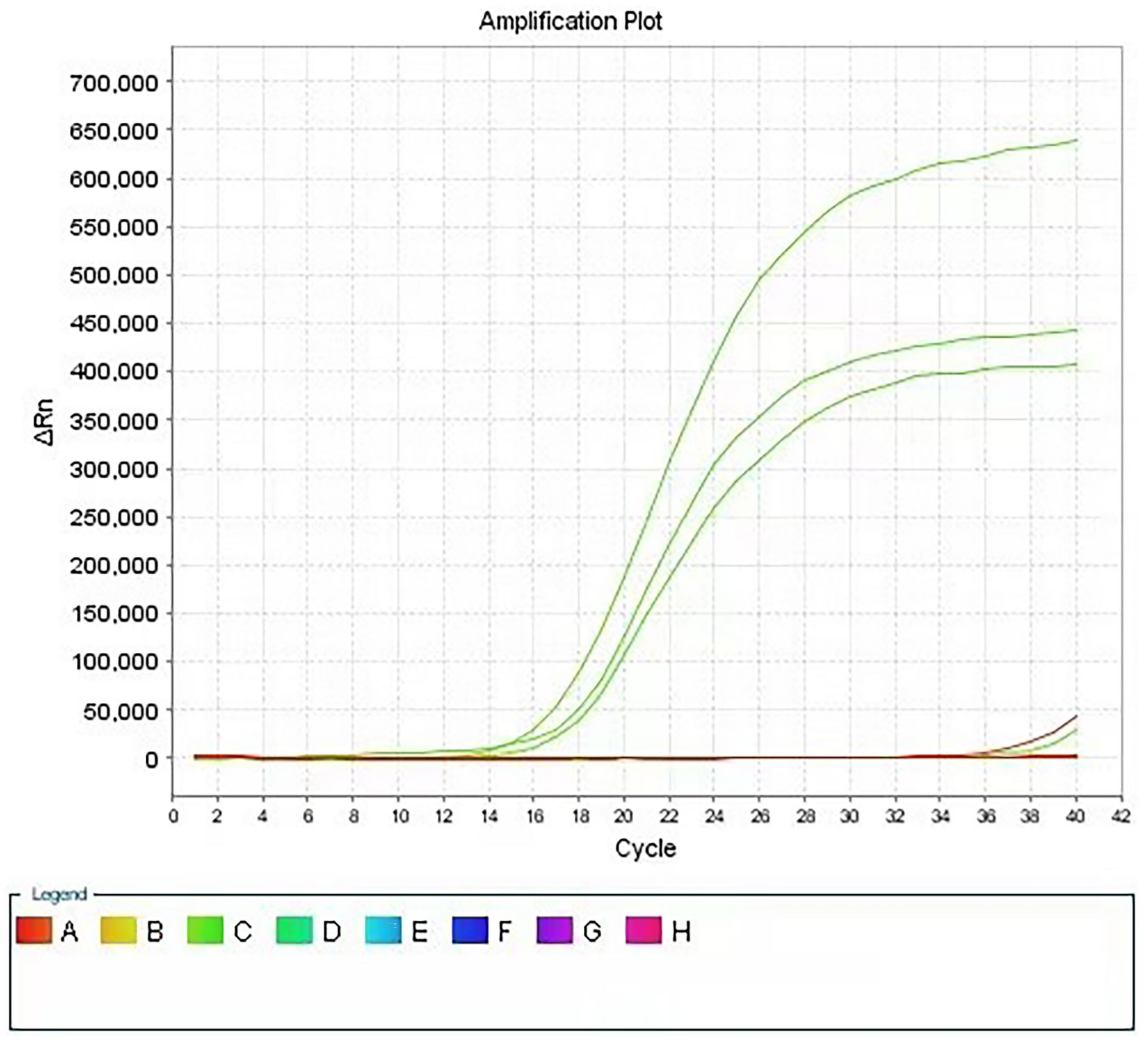
Amplification curve of PMA-qPCR assay for disinfectant products. Using specific primers for three indicator bacteria, real-time quantitative detection of three indicator bacteria was realized in the same reaction system.

Figure 1 shows the qPCR amplification curves of the three indicator bacteria treated with PMA after exposure to the test product. Compared with that of the control sample (three indicator bacteria in contact with PBS), the amplification curve of the experimental sample showed a significant backward shift, that is, a significant increase in the Ct value. This result indicates that PMA effectively differentiated the live and dead bacteria in the experimental sample, which enabled subsequent qPCR to successfully quantify the live bacteria of the three indicator bacteria in the sample and finally achieved rapid detection of the bacteriostatic properties of the product. More importantly, as shown in Figure 1, the simultaneous addition of the designed primers and probes for the three indicator bacteria to the qPCR system enabled the concurrent quantitative detection of three different indicator bacteria in the experimental samples. In total, the results show that this method can successfully detect the quantification of three indicator bacteria in one sample, which has not been reported yet.

**Figure 2:**
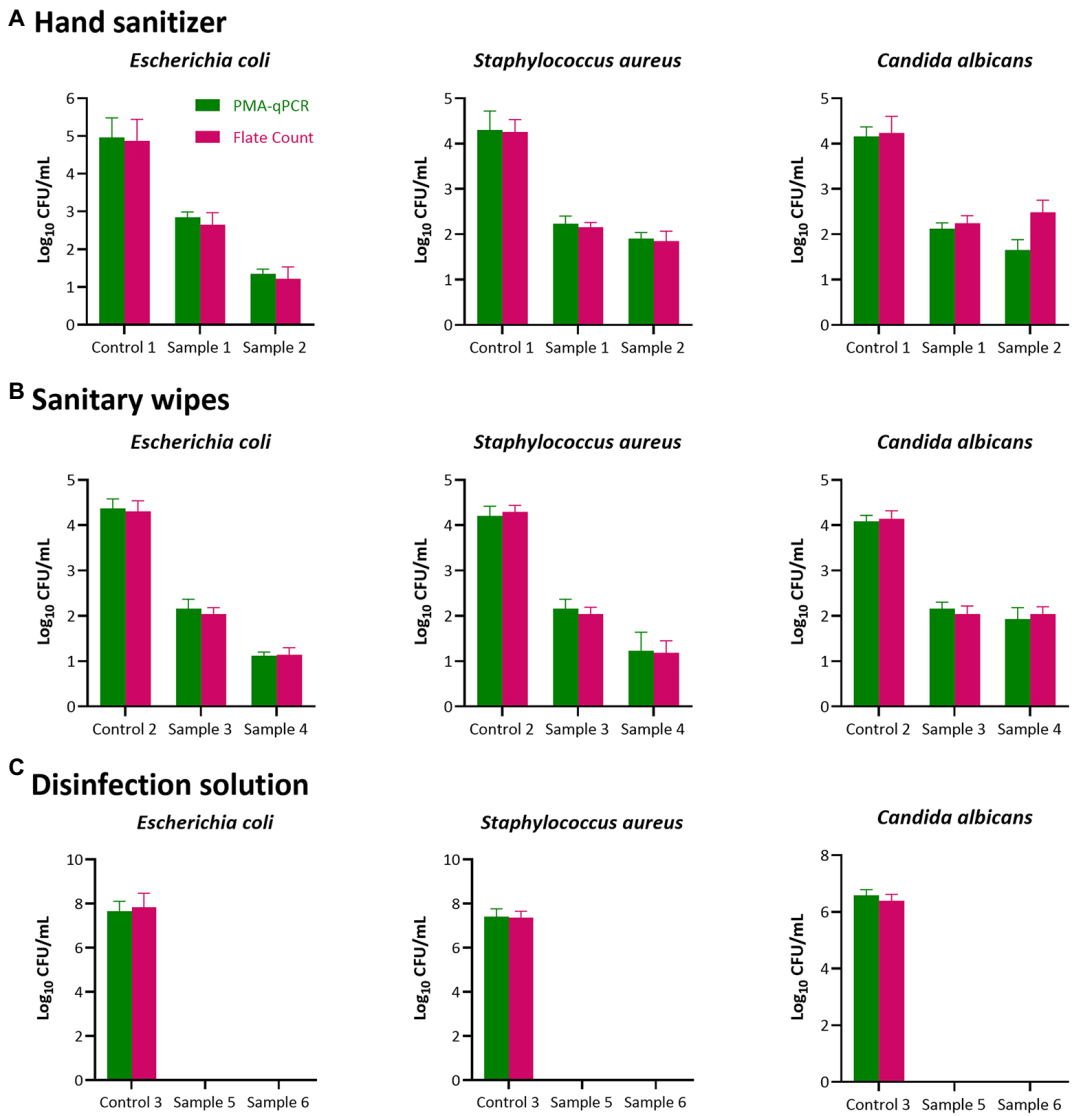
Antibacterial and Bacteriostatic activity test results of disinfectant products. Two individual methods (plate count in MRS agar and PMA-qPCR) were used to detect the antibacterial and bacteriostatic activity of the six disinfectants in three categories [**(A)**, Hand sanitizer; **(B)**, Sanitary wipes and **(C)**, Disinfection solutions)] Results represent the mean value of three repeats ± SD. No significant difference between the PMA-qPCR and plate counting methods in the quantitative detection of the three indicator bacteria after contact with disinfectants (*p >* 0.05).

In the present study, the established PMA-qPCR method was applied to test six disinfectants in three categories, hand sanitizers, sanitary wipes, and disinfection solutions, as shown in Figure 2. There was no significant difference between the PMA-qPCR and plate counting methods in the quantitative detection of the three indicator bacteria after contact with disinfectants. This result illustrates that the PMA-qPCR method established here is accurate and appropriate for detecting the antibacterial and bacteriostatic activity of disinfectants.

## Discussion

According to China Technical Standard For Disinfection 2002, the plate counting method is the national standard detection method for the numerical detection of viable microorganisms in disinfectants. This method is simple; however, test results depend on the growth of microorganisms, which takes approximately 48 h (bacteria) to 72 h (yeast). In addition, owing to the limitations of the culture medium and conditions, the plate counting method can only detect bacteria suitable for growth under the corresponding culture conditions. Many indicator bacteria in a sublethal state (VBNC) cannot form colonies in conventional culture media (Li et al., 2017; Golpayegani et al., 2019). Therefore, it is difficult to evaluate the number of indicator bacteria before and after sterilization using daily chemical disinfectants.

At the same time, antibacterial and bacteriostatic activity tests of disinfectants usually require two to three indicator bacteria: *S. aureus*, *E. coli*, and *C. albicans* (Jampilek, 2018). During detection, the same product to be tested needs to be applied to different indicator bacteria and the quantitative detection of each indicator bacteria after treatment. In conclusion, current national standard methods are unable to rapidly evaluate the antibacterial and bacteriostatic activity of disinfectants. To date, no other methods have been developed to detect the antibacterial and bacteriostatic activity of disinfectants.

Currently, PMA combined with qPCR is primarily used for microbial detection in food, medicine, and the environment. However, there are no reports on the antibacterial and bacteriostatic activity detection of disinfectants. The standard curve of PMA-qPCR was drawn by preparing the membrane-damaged bacteria of the target microorganism using the DNA from the membrane-damaged bacteria to conduct a gradient dilution of its living bacterial DNA, and then drawing it according to the Ct value of the qPCR reaction. Therefore, the preparation method of membrane-damaged bacteria is the key to distinguishing between live and dead bacteria of the target microorganism by PMA dye, and it also determines the accuracy of PMA-qPCR for the detection of living target microorganisms. Heating is the most commonly reported method for preparing membrane-damaged bacteria (Chen et al., 2011; Løvdal et al., 2011; Ditommaso et al., 2015). However, we showed that although the heating method had a good crushing effect on the three indicator bacteria, there was no colony growth on the corresponding plates after heat treatment. Moreover, the qPCR results (Table 4) showed that Ct values changed before and after treatment, especially for *S. aureus* and *C. albicans*, where the ΔCt values were 6.24 and 4.87 before and after heat treatment, respectively. These results revealed that the treatment caused different degrees of DNA damage in the three indicator bacteria and affected the amplification of the PCR reaction. Therefore, the heating method was not suitable for the preparation of membrane-damaged bacteria in this study. In addition, three indicator bacteria with membrane damage were prepared using the optimized homogenization method and analyzed using PMA treatment and qPCR. The results showed that the ΔCt values of qPCR before and after PMA treatment of the three indicator bacteria were 12.83, 12.73, and 11.10 for *E. coli*, *S. aureus*, and *C. albicans*, respectively (Table 6). PMA can clearly distinguish between living and membrane-damaged bacteria. It is worth noting that the preparation method of membrane-damaged bacteria in the current study can be applied to indicator bacteria for antibacterial and bacteriostatic activity detection of three disinfectants simultaneously, providing favorable conditions for the simultaneous detection of antibacterial and bacteriostatic activity of one disinfectant against multiple indicator bacteria.

Finally, the PMA-qPCR quantitative detection method established herein was used to rapidly detect the antibacterial and bacteriostatic activity of the six disinfectants in three categories. The quantitative detection results of living bacteria before and after contact with the indicator bacteria of each disinfectant was not significantly different from those of the plate counting method, and the detection time was shortened to 3 h compared to 48 ~ 72 h for the plate counting method (Figure 2). The present study markedly improves the detection efficiency of the antibacterial and bacteriostatic activity of disinfectants and their quality supervision efficiency (Drauch et al., 2020; Filipe et al., 2021). The PMA-qPCR method established here also provides the potential for detecting live mixed bacteria in complex samples.

## Conclusion

The current study is the first application of PMA-qPCR for the rapid detection of the antibacterial and bacteriostatic activity of disinfectants. There was no significant difference between the method established here and the plate counting method in the rapid detection of the antibacterial and bacteriostatic activity of the six disinfectants in the three categories, indicating the high accuracy of this method. In addition, this method inspects disinfectants in a reaction system concurrently to carry out quarantine on three commonly used indicator bacteria/antimicrobial resistance testing. Furthermore, it significantly simplifies the testing steps, reduces the testing time, improves the quality of disinfectant regulatory efficiency, and safeguards people’s health and safety.

## Data availability statement

The original contributions presented in the study are included in the article/supplementary material, further inquiries can be directed to the corresponding author.

## Author contributions

YL, SH, JZ, CZ, FH, YX, HQ, and YY participated in the design and discussion of the study. YL and SH carried out the experiments. YL, SH, and YY wrote the manuscript. JZ, CZ, FH, YX, HQ, and YY discussed, revised, and edited the manuscript. All authors have read and approved the final version to be published.

## Funding

This work was supported by the State Administration for Market Regulation Foundation of China [Grant number 2020MK136], and the Key Laboratory of Biotoxin Analysis and Assessment for State Market Regulation.

## Conflict of interest

The authors declare that the research was conducted in the absence of any commercial or financial relationships that could be construed as a potential conflict of interest.

## Publisher’s note

All claims expressed in this article are solely those of the authors and do not necessarily represent those of their affiliated organizations, or those of the publisher, the editors and the reviewers. Any product that may be evaluated in this article, or claim that may be made by its manufacturer, is not guaranteed or endorsed by the publisher.
